# Zoonoses, One Health and complexity: wicked problems and constructive conflict

**DOI:** 10.1098/rstb.2016.0171

**Published:** 2017-06-05

**Authors:** David Waltner-Toews

**Affiliations:** Community of Practice for Ecosystem Approaches to Health-Canada (CoPEH-Canada), Centre de Recherche Interdisciplinaire sur le bien-être, la santé, la société et l'environnement (CINBIOSE), Université du Québec à Montréal, Montreal, Quebec, Canada H3C 3P8

**Keywords:** conflicts, complexity, One Health, zoonoses, narratives, emerging infectious diseases

## Abstract

Infectious zoonoses emerge from complex interactions among social and ecological systems. Understanding this complexity requires the accommodation of multiple, often conflicting, perspectives and narratives, rooted in different value systems and temporal–spatial scales. Therefore, to be adaptive, successful and sustainable, One Health approaches necessarily entail conflicts among observers, practitioners and scholars. Nevertheless, these integrative approaches have, both implicitly and explicitly, tended to marginalize some perspectives and prioritize others, resulting in a kind of technocratic tyranny. An important function of One Health approaches should be to facilitate and manage those conflicts, rather than to impose solutions.

This article is part of the themed issue ‘One Health for a changing world: zoonoses, ecosystems and human well-being’.

## 1. Introduction

Since the 1980s, world health authorities have documented the emergence or re-emergence, and spread, regionally and/or globally, of emerging infectious diseases (EIDs). These EIDs, mostly zoonotic or emerging from animal reservoirs and adapting to human transmission, have included Acquired Immune Deficiency Syndrome (AIDS), some forms of malaria, bacterial food-borne diseases, Severe Acute Respiratory Syndrome (SARS), Q fever, influenzas (H1N1, H5N1), Ebola, Nipah and West Nile virus disease [1]. Each outbreak and epidemic can be characterized as a single case, attributable to specific causes and responses can be initiated using biomedical expertise and procedures. Treatments include triage, emergency medical care and delivery of appropriate drugs. Based on what was learned in that initial response, recommendations are made for prevention—usually using the same model which worked well in the emergency phase: the application of top-down technical expertise. For EIDs, these recommendations have tended to focus on better surveillance and vaccine development and delivery [2,3].

## 2. Causes of emergence: first responses

Considered within short time frames (weeks to months), and looking only at narrowly defined outcomes, such as stopping a disease, the results of these efforts have often been impressive. However, just as an acceleration and clustering in numbers of individual cases can be redefined at a larger scale as an epidemic or pandemic, so the accelerated outbreaks of EIDs comprise a pandemic of epidemics, emerging from deeper, systemic problems. Viewed from this perspective, the results of responses have been less than stellar, as more and more cases—that is, epidemics—keep walking through the door.

As the epidemics kept coming, some infectious disease experts worked to reduce the complexity of the problems to fit the theories and techniques with which they were most familiar, such as quantitative modelling, with a view to doing more of the same, only ‘better’ [1]. Other researchers have taken a broader view, identifying what were called drivers of EID emergence, rather than causes (as used in treating individual cases) or determinants (the term most often used by epidemiologists) of EIDs. A 1992 report by the Institute of Medicine in the USA, for instance, suggested that drivers of EIDs included human demographics and behaviour; technology and industry; economic development and land use; international travel and commerce; microbial adaptation and change and breakdown of public health measures [4]. Nevertheless, a 2012 review of progress in the 20 years since the original report noted the emergence of several new diseases, such as SARS and H5N1, and highlighted ‘genomics-associated advances in microbial detection and treatment, improved disease surveillance, and greater awareness of EIDs and the complicated variables that underlie emergence’ [5]. The review made no mention of initiatives to change global policies on such issues as industrial development, land use and trade.

Some of those larger drivers were being investigated, in what appeared to be a different universe, by those concerned with the social determinants of disease. Based on an extensive review of the literature, the authors of a 2008 World Health Organization (WHO) report announced that social injustice was ‘killing people on a grand scale’. The authors recommended that governments work to ‘improve daily living conditions, including the circumstances in which people are born, grow, live, work and age’; and ‘tackle the inequitable distribution of power, money and resources—the structural drivers of those conditions—globally, nationally and locally’. [6]. Other initiatives outside the EID community, but complementary to it, included the negotiation of Millennium Development Goals (MDGs) [7] and the more recent Sustainable Development Goals (SDGs) [8], as well as the health reports of the Intergovernmental Panel on Climate change [9], and the Millennium Ecosystem Assessment (MEA) [10]. The MEA used the term human well-being more or less synonymously with the 1948 WHO definition of health, which encompasses ‘complete physical, mental and social well-being and not merely the absence of disease or infirmity’ [11].

## 3. Integrative responses

Despite the good intentions, substantive links between those investigating, and responding to, EIDs as products of social, economic and political forces, and those viewing them through biomedical or ecological lenses, have been slow to emerge. Some health researchers and professionals, recognizing that many of the socio-economic and ecological drivers of disease are entangled, have attempted to integrate them into broad, systemic models, processes and frameworks.

Each of these integrative initiatives grew out of distinct scholarly cultures, each with their own languages, dialects, cultures and conferences. Academic researchers have been able to map out the linkages across temporal–spatial scales and disciplines necessary to address more fully the complexity of EIDs [12]. The authors of that paper—all scientists—argued that some form of transdisciplinarity was required but also acknowledged the institutional and logistical barriers to achieving such an approach. Ecosystem approaches to health (Ecohealth) grew out of a community of researchers focused on international development. Although drawing on quantitative epidemiological modelling, Ecohealth practitioners have tended to prefer more qualitative, community-based approaches, with health as one outcome of complex social-ecological changes [13]. One Health emerged from attempts to provide economic justifications for integrated animal–human initiatives and to coordinate the activities of trans-national organizations such as the WHO, the World Organisation for Animal Health (OIE), the World Bank and the Food and Agriculture Organization (FAO) [14,15]. Other integrative approaches, often involving some combination of modelling and narrative, have been proposed by researchers in the social sciences and the humanities [16–19]. These newer integrative approaches are solidly grounded in a vast body of scholarly literature, theory, practice and organizational experience of those working in business, ecology, policy, philosophy of science, social change and environmental management [20–23].

In general terms, the common intent of all these groups would appear to be unassailable—simultaneously to promote the health of people, other animals and the ecosystems we share. However, while attractive as a goal, such a transcendent, integrative notion of health has presented some intractable problems in practice.

Despite some dramatic successes in disease control, some of the major drivers of EIDs have worsened, and advocates for managing social determinants, ecological resilience, biodiversity and environmental conservation have had little success in influencing relevant national and international policies. Some might argue that EIDs, like many of the other problems we face—changing human demographics, climate change, heterogeneous food shortages and gluts, regional war, species extinctions and loss of biodiversity—are the products of an over-populated, over-consuming world, which itself is an outcome of earlier scientific and technical successes. If this is so, however, then this should give us pause: if scientists are so good at teasing out causes and effects, and predicting outcomes, why did not they see this coming? The European Environment Agency has reported on some aspects of this problem in two publications reviewing what they call ‘Late lessons from early warnings’. The first report, published in 2001, considers cases from 1896 to 2002 [24]. The authors of that report note that standard regulatory and scientific approaches do not handle the kinds of uncertainty and ignorance that are inherent in dealing with ‘complex, cumulative, synergistic or indirect effects’. The analysis of how the Bovine Spongiform Encephalopathy (BSE) epidemic unfolded is particularly relevant to EIDs, as it reveals the degree to which scientific advisors acquiesced to the government's reassuring narrative, constructed to maintain public and business stability, even when the evidence suggested other, more troubling, narratives. Some have referred to this tendency for scientific acquiescence to, or marginalization from, monetarily driven narratives as leading to ‘policy-based evidence’, that is, the converse of ‘evidence-based policy’ [25].

## 4. Problems of theory and practice

The stumbling partial successes of many global responses to EIDs can be attributed to the deification of certain biomedical, natural science and organizational narratives, and the marginalization of insights from social sciences and humanities.

In Cartesian (normal) science, one studies the parts and then adds them together to understand the whole. At small temporal and spatial scales, causal structures may appear to be linear: exposure to infected chickens preceded avian influenza cases in people. The focus is narrow, the path to success achieved by, race-horse-like, wearing blinders, peripheral vision (awareness of context) is denigrated as irrelevant and theories and values are assumed and poorly understood. As scholarly understanding of the spatial and temporal contexts for EIDs have expanded outwards to encompass regional and global pathways, and deepened into characterization of the molecular structures of agents and the proteins with which they interacted in people, some researchers attempted to create more complicated mathematical models. The idea is that one brings together ‘under one roof’ information about atoms, molecules, laboratories, homes, wildlife, farms, insects, men, women, kitchens, schools, communities, populations and landscapes—and relationships among all these. At some point, however, the models have become unworkable. The more precise they are, and the more variables they include, the more unwieldy and the less useful they become [26]. If models include more variables than well-defined independent equations, unique solutions cannot be obtained. Even at its best, systems modelling tends to focus on relatively stable relationships among variables and obscures the longer-term narratives and the values from which those relationships have emerged, and the instability of many relationships.

Ecosystems researchers and managers were some of the first to recognize this. Models work for some types of complicated problems, they concluded, like sending a man to the moon, but they do not provide much useful understanding of complex phenomena, like raising children, deciding what to do about genetically modified organisms (GMOs) or BSE or invasive species, or preventing EIDs [27,28]. As the MEA recognized, all attempts to model these complex eco-social phenomena have foundered on shoals of multiple scales, perspectives and knowledge systems [29].

Wicked problems arise in situations that can be defined from a variety of apparently incompatible perspectives. Since there is no definitive problem formulation, and scientific uncertainties confound all formulations, they cannot be resolved in any definitive manner. Solving one part of a wicked problem may exacerbate other parts. Health presents a set of wicked problems, with multiple possible, and sometimes contradictory, evidence-based solutions [30]. Public health initiatives that involve draining swamps or spraying pesticides may control a disease, but compromise other aspects of health related to environmental sustainability, livelihoods or nutrition. Those other outcomes may then feedback to undermine or even worsen the original, successfully achieved, outcome. Linear causal models and responses that work well within short temporal and spatial frames are inadequate for dealing with outcomes embedded in complex ecological and social relationships. To list drivers of EIDs as a list of independent variables is deceptive; they are entangled in complex spatial and temporal webs in which relationships change over time. There are no definitive experiments in such situations, where ‘facts are uncertain, values in dispute, stakes high and decisions urgent’ [31].

## 5. Complexity and narratives

If experiments and models are not up to the task, where can we turn? Some ecosystems researchers concluded that complexity, as manifest in wicked problems, are best investigated and understood using narratives, including diverse contending narratives [17,27,28]. The notion of using narratives to understand EIDs (as differentiated from merely communicating information pre-digested by experts) offers some possibilities for working through the current global swamp of contested facts, values and policies [17].

If narratives are such effective tools, why have EID researchers not used them more often? There are several reasons for this, having to do with both academic cultures and the nature and framings of the issues being addressed. The easiest answer for this oversight in the EID community is that the disciplines most familiar with investigating complex eco-social phenomena—political economy, anthropology, history, sociology, philosophy—are often dismissed as ‘soft’, ‘poor cousins’ to the natural and biomedical sciences. The latter are considered ‘hard’ science; the so-called ‘soft’ disciplines also suffer from neglect because, compared to natural sciences, with their standard rules and practices for replication and determining quality, they are much more technically and ethically challenging.

Nevertheless, faced with serious conundrums at the interface between science and policy, some researchers have looked to the narratives and analytic methods of the humanities and social sciences for insight [17,32–35]. Some literary analogies would be novels focused on a single character (a virus, person), those focused on family dynamics (outbreak level) or those describing the broad sweep of history (epidemics and pandemics). Narratives appear to be more adept than models at accommodating varieties of diverse evidence, on different temporal and spatial scales, and drawing from laboratory research, political analyses, economics, geographic information systems and quantitative models to indigenous story-telling, music, poetry and photography [27,33].

However, we are now faced with a different challenge. Each of these explanations of EIDs, based on different cultures and values and different notions of what qualifies as evidence, offers possibilities for resolution of the problem that may exacerbate outcomes for some stakeholders even as they mitigate others for other stakeholders. They thus would seem to create new problems (conflicting narratives) even as they solve others (accommodating multiple kinds of evidence).

Why does this happen? Narratives, because they are observer dependent, expose what in many mathematical and epidemiological models is hidden or obfuscated: issues of values and power relationships. In an introduction to a 1995 re-consideration of JBS Haldane's famous 1923 ‘Daedalus, or Science in the Future’, Nobel Laureate Joshua Lederberg argued that ‘science is bereft of deontology: it cannot tell why one should be interested in science or anything else’ [36, p. ix]. The fundamental point here is that all researchers and practitioners frame their work in such a way that it accords with their personal values, and those values can be contested, but are not testable. Furthermore, if the context changes, solutions may change; with a scientific assumption that truth is univocal and universal and best understood by well-funded scientists, the notion that ‘truths’ can change depending on context and narrative seems ‘soft’ and anti-scientific. Yet, in the context of a ‘real world’ of evolving species, changing cultures and unstable climates, scientific attitudes routinely remain unexamined and muddled.

One outcome of this unexamined nexus of science and values is that political and public health leaders make decisions based on a combination of how convincing particular narratives are, how well they fit into the larger cultural narrative that gives them their power and the practicality of solutions suggested by the narrative [24]. These are then justified based on selected evidence. In the twenty-first century, the narratives of health, disease and development have been primarily narratives that justify centralization of power in the name of an unexamined greater good, which usually means the good of large trans-national institutions, corporations and their investors [17]. Certain kinds of expertise (in academic terms one might think of political economy, anthropology, history, philosophy) and certain kinds of outcomes (economic and social inequity, ecological and community resilience) are marginalized and largely dismissed as being irrelevant, non-scientific, under-developed, primitive. Until very recently, the political, economic and value-laden character of this dominant narrative remained largely unexamined [34,35,37].

## 6. Perspectives, values and post-normal science

It is not enough to be clear about our own and others’ value assumptions. Scholars and policy-makers also make assumptions about how the world works. What investigators call ecosystems are descriptions of particular aspects of nature, dependent on both the observer (hence the values), and what is being observed, which is based on some model of how nature ‘works’ [38]. For many scientists, this assumed mental model of the world is never articulated, because being like fish in water, the nature of the water that gives us life is too obvious to comment on.

One Health advocates may articulate what appear to be globally held values—as reflected perhaps in the MDGs and SDGs. However, if the One World to which One Health is being applied is understood as one gigantic complicated system, a computer perhaps, then ‘problems’ such as EIDs are seen to be merely technical, solved by more data, innovations in technology and deft or aggressive organizational footwork. The message from history of EIDs, and research into them, is that this approach has serious flaws that cannot simply be readily solved by better surveillance, vaccines, drugs and military-type rapid response teams in white bio-security suits. EIDs are symptoms of wicked problems embedded in complex social-ecological feedbacks, characterized by changing inequalities of social and economic power, well-intentioned ecological destruction, repression of eco-social diversity in the name of better healthcare, colonial attitudes and paternalistic environmental management.

One could site many examples—from the explosive pandemics of Ebola and SARS to the slower and more persistent spread of Lyme disease and food-borne illnesses—where apparently unrelated events in land-use planning, travel, agricultural policy and dietary preferences have resulted in unexpected outcomes. Some of the best-documented narratives are those related to emergence of highly pathogenic H5N1.

In 2006, an editorial in the *New England Journal of Medicine* suggested that, were severe pandemic influenza (H5N1) to invade the USA, two-thirds of the population could be infected, two million could die, medical costs would be hundreds of billions of dollars and the gross domestic product would drop by 5% [2]. Newspaper headlines at the time reflected a war-like stance against this apparently unexpected menace.

In Europe and Russia, concern that migrating waterfowl were acting as vectors for this ‘deadly’ virus prompted hunters to offer to shoot incoming birds. By 2006, defence-like early detection systems were in place in Canada, the USA and several other countries [39,40]. Their purpose was to monitor for incursions of the pandemic virus through migratory waterfowl.

Let us for a moment consider the narratives from which these events emerged.

## 7. The chicken

The wild male jungle fowl, primary progenitor of the world's domestic poultry, is a magnificent bird, his wattles full, fleshy, his pink cape flowing from the crown, over his back, in brilliant bronze, rust and gold, ending in a ruff of white. His lower chest and fountaining tail feathers shimmer in the early morning sunlight from deep black to blue to green. His sharp black eye catches yours and holds it. This is not a bird to quietly back down from a challenge [41].

Originally domesticated for cockfighting sometime between 8 and 10 000 bc, poultry had migrated westward from India into Persia and Africa by 2000 bc and into Greece by 500 bc. By about 200 ad, the Romans were using chickens as religious augurs and had developed commercial poultry rearing for food. Chickens arrived in the Americas a century before Columbus, probably from Polynesia [42]. During the spread of the Jungle Fowl to most inhabited portions of the globe, it carried with it not only its wild spirit but also a host of viruses and bacteria inhabiting its intestines.

## 8. The waterfowl narrative

Waterfowl were domesticated even before jungle fowl, probably in the Middle East—‘cradle of civilization’—and from there spread throughout the world. Because these birds need standing water, agricultural rearing of ducks tended to be restricted to countries where agriculture involved a lot of water, as in rice paddy culture. In China, large-scale duck farms are reported as early as the fifth century bc.

As with chickens, the ducks came equipped with their own microbiomes. The intestines of waterfowl are the primordial home for all 16 known subtypes of the influenza A virus [43]. Although inherently unstable, these influenza viruses have been in evolutionary equilibrium and have not normally caused disease in their natural hosts.

Historically, the public health impacts of small-scale waterfowl and poultry rearing have tended to be localized and minimal. However, when waterfowl are intensively mixed with chickens and pigs and people in close quarters, as they are in many parts of South East Asia, novel opportunities for the viruses are created. Under these conditions, their instability allows them to mix and match genetic material from various sources. Pigs have receptors for both human and avian influenza viruses, so serve as a mixing bowl for annual influenza pandemics that annually infect about three to five million people and result in up to half a million deaths [44].

Since the late 1990s, concern has focused on direct transmission of influenza viruses between birds and people. Since poultry and waterfowl influenza viruses tend to attach low in the human respiratory tract, they are more difficult to contract; but among those so infected, the disease is worse. Of even greater concern: the newly emerging influenza viruses were now killing the birds that hosted them, and with whom they have historically lived in balance.

## 9. The food security narrative

In the early seventeenth century, King Henry IV of France announced that ‘If God keeps me, I will make sure that no peasant in my realm will lack the means to have a chicken in the pot on Sunday!’ [45]. This has become a recurrent dream and promise of many political leaders since that time. By the late twentieth century, citizens of countries established by the European diaspora (North America, Australia, New Zealand, etc.), as well as people living in parts of East and Southeast Asia, had exceeded the king's wildest fantasies: we could put a chicken in every pot every day.

According to FAO statistics, in 1961, the world population of domestic chickens numbered about four billion. By 2013, that number had grown to about 20 billion chickens. In the twenty-first century, chickens are being grown, trucked, shipped and fried as fast as technology allows. Domestic duck populations increased from fewer than 200 000 to more than a billion during the same time period [46]. Who would have thought that so many people on this planet could be fed with such apparent ease?

## 10. How was this accomplished?

In the first instance, we should recognize that there are two main parts to Henry IV's dream. Although the dream has been marketed as being primarily an agricultural and scientific challenge (the capacity of farmers to grow more birds), the ability of peasants to buy those birds—an economic problem—is equally important. One possible solution to the economic issue would be some kind of wealth redistribution, to increase the income of workers and peasants, so that they could afford to pay the true costs associated with food production.

However, this socio-economic narrative and possible strategic solutions that arise from it have been marginalized and treated as politically disruptive to the smooth functioning of ‘scientifically based’ industrial agriculture.

It is worth examining both this type of agriculture and the science that has served it so well. Much of science as we have come to understand it emerged from the works of natural philosophers and scientists in the seventeenth century, who sought to ground human knowledge in observation of natural phenomena, rather than on received wisdom of scholars.

One strand of this science—the one that came to dominate much of what we call science today, and which we have come to consider orthodox—argued that one can and should understand and master nature by dividing it into smaller and smaller parts, and studying those parts [47]. The dominance of this kind of science coincided with, and was driven by, the narrative of industrialization and progress [25].

Business leaders in the industrial revolution understood that if one manufactured the parts separately, and then assembled them in factories, one could produce commodities in such a way that even ‘common workers’ could afford them. Applying this to poultry production (note the language), the economies of specialization and scale suggested that a ‘chicken in every pot’ might be achieved by putting many thousands of birds into very large barns, with genetic, feed and housing inputs manufactured in separate factories. One factor that kept poultry farmers from immediately adopting mass production of chickens was that the birds need access to sunlight in order to synthesize vitamin D necessary for bone growth and egg production. Another constraint was the fact that, when animals are crowded together, they are more likely to shed higher numbers of bacteria, which then put flocks at greater risk for spreading epidemic diseases [48]. In the period after World War II, however, orthodox natural scientific methods came to the rescue: agricultural scientists were able to formulate feeds fortified with vitamins and antibiotics. These breakthroughs finally made it possible to realize King Henry IV's dream: a chicken in every pot.

Once these breakthroughs occurred, agribusinesses were able to quickly scale up and globalize. In the 1990s, the agricultural industry responded to rapidly urbanizing populations and changing food demands in the developing world with the same tactics they had used for the past century in Europe and North America—only faster. By drawing on advances in genetic manipulation and intensive, selective breeding, they transformed the jungle fowl into something that could be grown faster, more uniformly, and, by some standards, more efficiently than any bird in history. Now there was a chicken of identical size in every pot. And if the genetic stock, the feed and sometimes even the buildings were exported to developing countries, and low-priced local labour used in place of under-priced fossil fuel, this feat could be accomplished just about anywhere in the world.

## 11. Unintended consequences of orthodox scientific solutions

But what happens if the narratives we have constructed are simplifications of complex webs of interactions and feedbacks, and not simply a series of independent problems to be solved? Economies of scale have some obvious direct benefits; they enable the production of large volumes of chicken, which can, given current market rules and constraints, be sold at relatively low prices. But they also rely on the enforcement of a centrally controlled, top-down, vertically integrated management system; that is, they rely on a stable form of structural inequality, with far-reaching ecological and social consequences. These unintended consequences then looped back, over time, with large adverse human health consequences. The emergence of diseases associated with such agents as H5N1 and salmonellosis is not, if viewed through a lens of complexity, surprising; the very characteristics that facilitated large volumes and global distribution also facilitated the emergence of zoonotic diseases.

Although not explicitly characterized as such, the emergence of H1N1 in Mexican pig populations and its spillover into humans in 2009 reflected a compact example of the same pattern [49]. Pigs were raised in large-scale operations in Mexico for the North American market for reasons similar to those used for large-scale poultry production. Mexico (and many other countries) offer low-cost labour and somewhat relaxed labour laws. An infected farm worker, receiving low wages and with no paid sick leave or health insurance, would have every incentive to go to work even when seriously ill. Whether the infection entered the pig population from infected people, or whether it emerged in the pigs and then spread to the farm workers and their families is largely irrelevant. Either way, under these inequitable social and labour conditions, the virus would have been cycled back into the pigs in a positive feedback cycle. An obvious measure to prevent the spread of the virus would have been for all countries importing pigs from these farms to require them to have paid sick leave and health insurance for their workers and to meet minimum standards of hygiene.

‘Problems’ associated with human labour of that type have sometimes been avoided in poultry production largely by replacing low-paid people with computer systems. This prevents the H1N1 effect but has other unintended consequences with regard to rural unemployment, energy use and food-borne diseases. Economies of scale in poultry production require large, climate-controlled barns, which require a stable, low-cost source of energy. These have been managed until now through financial subsidies to companies mining fossil fuels.

Chicken feed requires a protein source. Pillaging—no gentler word will suffice here—of wild fish stocks (particularly anchovies) off the coast of Peru and clearing Brazilian forests to grow soybeans provide two excellent protein sources. Corn, with its huge subsidies in the USA, provides another important feed ingredient.

The antibiotics used in the feed have exposed the poultry microbiomes to intense evolutionary pressure. A wide variety of studies over the past 40 years have identified causal relationships between antibiotic use on farms and the emergence of antibiotic-resistant bacteria in animals and people. To the surprise of economists and agribusiness leaders, but certainly not of evolutionary biologists, antibiotic-resistant bacteria travelled through national and global trade networks and soon attained pandemic levels in poultry, eggs and feeds [50–52].

Alongside this, and often neglected in discussions of food security or disease, is the matter of agricultural waste. Nineteen billion birds produce a lot of manure: about 500 million tonnes per year [53]. Add to this both the ‘normal’ pre-slaughter mortality rate of a few per cent, and the inedible offal from butchering, and it should be clear that the industrialization of poultry production somehow has to deal with a lot of organic waste. Within integrated livestock systems, some of the manure is recycled into cattle feeds, or used as fertilizer, and the other organic materials are added to livestock and pet feeds.

But the manageability of this system, based on extreme economies of scale, is illusory. Quite apart from the impacts already described, industrial poultry production is responsible for high rates of food-borne infection that in some cases—such as *Salmonella enterica* and *Campylobacter* spp.—have become an unavoidable ‘cost’ of production. In the UK, levels of *Campylobacter* contamination of poultry carcasses is so high that in 2015 the UK Food Standards Agency recommended that consumer should not wash raw chicken, but cook it thoroughly in its unwashed state [54]. The over-application of chicken manure on land in the USA has led to runoff into nearby waterways, and explosive outbreaks of the toxic dinoflagellate *Pfiesteria piscicida*, which in turn have killed millions of fish and sickened people who came into contact with it [55]. When the increases in domestic ducks and incursions into landscapes frequented by wild waterfowl are added to the mix, there is also a greatly increased likelihood that bacteria and viruses will sooner or later find their way to new host species—as is already the case with the avian influenza viruses [56,57].

In 1996, a precursor of the H5N1 virus killed some geese in South China. Few health practitioners paid much attention. Then the virus picked up some gene fragments from quail and ducks, spread to the poultry markets in Hong Kong, and made the leap over to people; it killed six of 18 people who were infected. Mass killing of all the domestic poultry in Hong Kong temporarily stopped the problem, but the virus continued to infect ducks and geese. It also continued to evolve. In late 2002, a new more lethal variation of the virus killed off most of the waterfowl in Hong Kong nature parks and spread through Vietnam, Thailand, Indonesia, Cambodia, Laos, China and Malaysia. Not only was it making birds sick and killing them, now it was also infecting cats and ferrets and, finally, people [56,57].

In May and June of 2005, one of the new variants of H5N1 killed more than 5000 wild bar-headed geese, gulls and ducks in Qinghai Lake, in China. Before they died, the affected birds developed neurological problems [58]. Researchers worried that migratory birds would carry the virus to India, Europe, the Middle East and Africa. This has since happened; but to what extent the world trade in poultry, both official and unofficial, might also be responsible remains an open question.

What is certain, however, is that a highly contagious strain of H5N1 can now be reached in only a few mutations. Such a strain could allow airborne transmission between mammals and in this way trigger a major pandemic. In fact such a strain already exists, thanks to laboratory scientists at University of Wisconsin-Madison who recently created a deadly variant of the influenza virus able to outsmart the human immune system [59]. The somewhat nebulous justification for this work is that it enables laboratory researchers to better understand such viruses and how to attack them. Stephen King in his 1978 horror/fantasy novel ‘The Stand’ envisioned the global apocalypse that would ensue were such a virus to escape from the laboratory. Many of us hope it will not come to that.

## 12. Complexity comes home to roost

Why did these unintended consequences occur? Global narratives—often assumed but not articulated by orthodox scientists—are woven from particular perspectives by experts who believe that science provide as absolute Truth, and that they, as experts, have ‘the answer’.

Instead of separating the challenges of production from those of affordability, analysing them and proposing complementary, sustainable solutions, the global narrative that came to dominate food security policies has been that economics of scale on farms combined with networks of international trade would solve all the important social problems; unintended consequences—emerging diseases, social and economic inequity, environmental degradation, species extinction—could be dealt with as necessary but unfortunate side effects. However, without an understanding of how multiple narratives, deeply rooted in different value systems and inequities of power, interact, these side effects are undermining our ability to negotiate our way through a series of global catastrophes.

The side effects, rather than being easily managed, have had the effect of accentuating evidence-based counter-narratives, inequalities in the system, magnifying environmental impacts and shifting economic and disease risks from agribusiness owners to farmers and consumers. Economies of scale are needed from ‘farm to fork’ so as to keep the chicken affordable for poor people and allegedly to better promote food safety, although there is no evidence that this is true. In order to ‘sustain’ economies of scale and keep shipping costs down, agribusinesses require low-cost energy and feed sources and/or low-paid labour. Energy supplies were achieved through subsidized fossil fuel production. Low-cost labour has been achieved by underpaying agricultural and food system workers, many of whom are of uncertain legal status, with little societal protection.

But not only have these extreme economies of scale had social and environmental costs, they also—given increases in human population, increased pockets of wealth and changes in diets to incorporate more meat protein—have required expansion into ever more habitats (energy production, large farms). This expansion into new habitats has been magnified by the concomitant increases of domestic duck production, which has led, in turn, to more intense interactions between wild and domestic fowl, and thence to the emergence of new strains of influenza.

Yet few animal science researchers, standing in their hazard suits and masks in a conventional chicken broiler barn, have made the mental connections between poultry rearing and ducks flying overhead, or between agribusiness and viruses and bacteria for which the ducks are a quiet, accessible vehicle for long-distance air travel. Nor have the linear narratives and logic of orthodox science been able to make apparent and manage the connections between increasing poultry production, systems of energy production, distribution and use, jungle clearance in Brazil (to grow soy protein), disappearing fish stocks in Peru (for fishmeal) and the even larger issues related to global changes in climate and biodiversity.

It is a grievous mistake to imagine that pandemics can be understood and managed by studying the pieces separately (viruses, birds, pigs, people). To understand the challenges of learning to live with diverse microbial populations, we need to re-imagine the world in deeper, more complex, more evidence-based ecosystem terms. It is one thing to document in detail the cellular and biochemical structure of dead ducks in a marsh in Saskatchewan, as well as, more recently, the microbiomes they carry, or those of dead chickens in a barn in British Columbia. It is quite another thing to understand the relationships among multiple species at multiple scales.

One counter-narrative might be that, because of our flawed assumptions about nature and an unwillingness to examine or even acknowledge values, political, economic and gender power differentials, the real costs of producing low-cost chicken are being paid in economic subsidies to fossil fuels and corn, in lost biodiversity in Brazil and in the oceans, and in urgent adaptations to dramatic, unstable climate change. They are mainly being paid by impoverished farmers and food industry workers; by millions of non-human species whom we abuse (in the system); by wild species whose habitats we are invading and destroying; by our grandchildren, whose choices are increasingly constrained; by already underfunded healthcare and public health systems and of course by the victims of consequent diseases.

Re-formulating this counter-narrative, one might suggest that supply-side, ecologically grounded livestock production is possible, but because this internalizes wider environmental and health costs, the meat and eggs they produce appear more expensive to the consumer. Certainly, the cost at the grocery store is higher for ecologically based farming. Those food costs could be off-set by variations of wealth distribution that range from traditional kin-sharing networks, cooperatives and voluntary associations to broader social safety nets, welfare states, social democratic programmes and guaranteed incomes. The alternatives should be seen as essential elements in a globally sustainable programme that accommodates food security, health (in its broad WHO definition) and ecological resilience.

## 13. Constructive conflict: a way forward

Acknowledging the alternative narratives requires that one acknowledge as well that one is faced with trade-offs and continual tensions. Policy actions will need to be renegotiated as situations change. This is the nature of complex reality, an understanding of reality based on observation of multiple strands of real-time events rather than fabricated from data in a computer model or laboratory [60]. For these ‘post normal’ questions, there will forever be scientific, scholarly, cultural, political and economic conflicts; they cannot, by definition, be resolved by gathering more data. Certainly the kinds of screaming fits that otherwise serious scholars have engaged in over, for instance, climate change, GMOs and BSE would seem to be not only undignified but undermine any serious confidence the general public might have in scientific research and scholarship. And circling the academic wagons into the more than 7000 scholarly journals currently being published is a kind of retreat. Yet scholars interested in policy applications, and with commitments to One Health, have obligations to engage with the conflicts and contradictions generated by their work and its relationships to policy.

How then can we move ahead? One health and Ecohealth practitioners and scholars are in a unique position, situated at the interface between the local and global, and between the natural world and the increasingly widespread urban, constructed world of people. One option would be to create scholarly and public spaces for managing constructive, high-quality conflicts. With leadership through One Health-Ecohealth platforms or alliances, if supported by sufficient funders, and one or more internationally recognized journals in sciences, humanities and social sciences, new spaces for creative, constructive, high-quality conflict could be provided.

The mandate of such spaces would be to:

1. Characterize the relevant scientific and policy issues requiring constructive conflict.
2. Create safe forums for articulating and debating issues where ‘facts are uncertain, values in dispute, stakes high and decisions urgent’.
3. Articulate possible outcomes (and to whom they might be ‘acceptable’), even if they are conflicting.
4. Create venues where trade-offs can be characterized.
5. Negotiate optimal outcomes and clear avenues for adaptation and change.
6. Identify appropriate (multi-level) organizational structures for implementing.

Science at its best is a way to share experience, to offer and evaluate alternative explanations, to project future possibilities based on past experiences. If complexity is taken seriously, and not just as a technical modelling issue, then notions of expertise, and monitoring, and facts, and the role of scientists and scholars, all change in some fundamental ways. As the late ecosystems scholar James Kay asserted:

Investigators into complexity do not seek prediction, control, right answers and efficiency. These are not sensible goals under conditions of complexity. Rather, the investigators seek understanding, adaptability and resilience. Scientific inquiry, more than ever, becomes an act of collaborative learning and knowledge integration. The role of the expert shifts from problem solving to an exploration of possibilities, from giving correct advice to sharing information about options and trade-offs. Those who cling to being the old sort of expert in fact lose their expertise.

Because there is no correct answer and no definitive perspective, decision-making under complexity will require new institutional arrangements, and broad public participation [61, p. 80].

In the end, just as there is no single value one can place on poultry, there is no generic EID, and there will be no single solution to the challenges of producing sufficient food on a sustainable basis, and to prevent the emergence and spread of infectious diseases. There will always be conflicts. It is in our human solidarity, and the ways in which scholars and policy-makers manage those conflicts, that the maturity of our science, and indeed of our civilization, is reflected [62].
